# Evolution and Therapeutic Approaches in Adolescents Hospitalized in a Psychiatric Unit : A 12-Month Study

**DOI:** 10.1192/j.eurpsy.2025.1131

**Published:** 2025-08-26

**Authors:** A. Gharbia, D. Mezri, S. Boudriga, G. Ouertani, U. Ouali, A. Aissa, R. Jomli

**Affiliations:** Avicenne, Razi hospital, Mannouba, Tunisia

## Abstract

**Introduction:**

As mental health challenges among adolescents continue to rise, hospitalized Adolescents in psychiatric units often face complex and evolving challenge.A lot of current research aims to optimize treatment strategies for this vulnerable population.

**Objectives:**

This study aims to explore evolution and therapeutic approaches of adolescents hospitalized for the first time in the psychiatric department.

**Methods:**

It’s a retrospective and descriptive study covering the files of patients who consulted the Avicenna Psychiatry department of Razi hospital aged under 19, during the year 2023.

The collection of anamnestic and clinical data was established using a pre-established form.

**Results:**

Our study included 24 patients aged between 16 and 18 years, with a mean age of 17 years, comprising 13 males and 11 females.

Patient follow-up in our service was regular in 58.33% of cases, while 20.83% of patients were lost to follow-up.

Psychotherapy was recommended in most cases, and medication was prescribed in 100% of cases, with only 58.3% adhering to the treatment. Relapse with rehospitalization was observed in 45.8% of cases, with a range of 1 to 35 readmissions. Parents of these patients were cooperative and receptive to the care in 62.5% of cases, while 12.5% of parents remained rejecting.

Clinical outcomes showed improvement in 33.3% of cases and worsening of symptoms in 45.8% of cases.

**Conclusions:**

Early and individualized therapeutic planning, in collaboration with family and social services can play a crucial role in preventing future hospitalization.

**Disclosure of Interest:**

None Declared

